# Virtual Reality to Improve Sleep Quality in Patients Suffering from Painful Diabetic Polyneuropathy: A Proof of Concept Study

**DOI:** 10.3390/jcm13237163

**Published:** 2024-11-26

**Authors:** Lisa Goudman, Ann De Smedt, Julie Jansen, Maxime Billot, Manuel Roulaud, Philippe Rigoard, Maarten Moens

**Affiliations:** 1 STIMULUS Research Group, Vrije Universiteit Brussel, Laarbeeklaan 103, 1090 Brussels, Belgium; 2 Cluster Neurosciences, Center for Neurosciences (C4N), Vrije Universiteit Brussel, Laarbeeklaan 103, 1090 Brussels, Belgium; 3 Department of Neurosurgery, Universitair Ziekenhuis Brussel, Laarbeeklaan 101, 1090 Brussels, Belgium; 4 Pain in Motion (PAIN) Research Group, Department of Physiotherapy, Human Physiology and Anatomy, Faculty of Physical Education and Physiotherapy, Vrije Universiteit Brussel, Laarbeeklaan 103, 1090 Brussels, Belgium; 5 Research Foundation—Flanders (FWO), 1090 Brussels, Belgium; 6 Department of Physical Medicine and Rehabilitation, Universitair Ziekenhuis Brussel, Laarbeeklaan 101, 1090 Brussels, Belgium; 7 CHU de Poitiers, PRISMATICS Lab (Predictive Research in Spine/Neuromodulation Management and Thoracic Innovation/Cardiac Surgery), F-86000 Poitiers, France; 8 CHU de Poitiers, service de Neurochirurgie du rachis, chirurgie de la douleur et du handicap, F-86000 Poitiers, France; 9 Pprime Institute UPR 3346, CNRS, ISAE-ENSMA, Université de Poitiers, F-86000 Poitiers, France; 10 Department of Radiology, Universitair Ziekenhuis Brussel, Laarbeeklaan 101, 1090 Brussels, Belgium

**Keywords:** immersive technologies, diabetes, chronic pain, behavioral therapy, biopsychosocial treatment, patient education

## Abstract

**Background/Objectives**: Sleep disturbance is often observed in the context of chronic pain. We hypothesize that, by providing an immersive Virtual Reality (VR) experience with a serious game to chronic pain patients an hour before bedtime, attention can be diverted from the pain condition, consequently leading to improved sleep quality. The aim is to evaluate the efficacy of VR compared to usual care in reducing the number of awakenings during the night and increasing sleep efficiency in patients suffering from painful diabetic polyneuropathy (PDPN). **Methods**: Eight patients with PDPN were randomized to either two weeks of VR or two weeks of usual care, followed by a cross-over. The primary outcome measurements were sleep efficiency and number of awakenings during the night. As secondary outcomes, self-reported sleep quality, insomnia, pain catastrophizing, anxiety, depression, pain intensity, side effects and impression of change were evaluated. **Results**: Data of seven patients were analysed. Actigraphy data, self-reported sleep quality, insomnia, pain catastrophizing, anxiety, depression and pain intensity scores did not differ between usual care and VR. As for impression of change, more patients improved after VR compared to usual care (V = 21, *p* = 0.03). **Conclusions**: A 2-week period of pain neuroscience education through VR did not result in increased sleep efficiency or fewer awakenings compared to usual care in patients with PDPN. These pilot results indicate that patients subjectively experience an improvement, yet this is not substantiated by either self-reported or objective measurements.

## 1. Introduction

Diabetes mellitus truly is a disease of the present day and age, posing a health concern for many countries in the world [1]. In 2017, about 425 million adults were living with diabetes mellitus globally, a number that is projected to rise to 629 million by 2045, according to the International Diabetic Federation [1]. Diabetic polyneuropathy is a serious complication and consists of a symmetrical, length-dependent polyneuropathy [2], characterised by simultaneous malfunction of many peripheral nerves, leading to various symptoms, among which are prickling, tingling, burning, itching and tight sensations [3]. The prevalence of diabetic polyneuropathy varies between 26% and 50%, depending on different assessment methods and definitions [4,5]. In patients recently diagnosed with type 2 diabetes, a prevalence of 18% was revealed for diabetic polyneuropathy, and the prevalence of painful diabetic polyneuropathy (PDPN) was revealed in 10% [5]. This chronic pain condition negatively influences various aspects of health-related quality of life including, but not limited to, disability, sleep quality and mood [6].

Unfortunately, PDPN still remains inadequately diagnosed and treated [7]. Based on international expert consensus recommendations, management of PDPN includes three cornerstones: (i) lifestyle modification, optimal diabetes mellitus treatment aimed at near-normoglycemia, and multifactorial cardiovascular risk interventions; (ii) pathogenetically oriented pharmacotherapy; and (iii) symptomatic treatment of neuropathic pain, including analgesic pharmacotherapy and non-pharmacological options [8]. In more detail, gabapentinoids, serotonin–norepinephrine reuptake inhibitors, sodium channel blockers, and SNRI/opioid dual mechanism agents have medium effect sizes on pain reduction, while tricyclic antidepressants demonstrated a large effect size [9]. Some of these medications (e.g., tricyclic antidepressants, serotonin–norepinephrine reuptake inhibitors) may also have a beneficial effect on sleep [9]. A trial of medication from a different class should be provided when patients do not achieve meaningful improvement or experience significant adverse effects with initial therapeutic classes [9]. To be able to reduce opioid use, which is not recommended for this patient population [9], and potential side effects of different treatment classes, patients would benefit from additional non-pharmacological interventions [10].

Sleep disturbance is one of the critical aspects significantly impacted by the presence of chronic pain, leading to an exacerbation of the negative impact on overall well-being [11]. A previous study in patients attending a primary healthcare center revealed that 71.8% of patients with chronic pain reported sleep problems [12]. Specifically in the context of diabetic neuropathy, considerable sleep impairment is reported [13,14,15], whereby patients with diabetes are more likely to have sleep disorders [16,17]. The main reason for sleep problems in people with diabetes is neuropathy [18]. Since pain has a biopsychosocial character, psychological therapies and behavioral medicine should also be applied to treat patients with PDPN via a holistic approach [19]. One such treatment option is pain neuroscience education (PNE), which has gained attention in a broad variety of chronic pain management settings, such as post-surgical settings, cancer populations, chronic musculoskeletal settings or paediatric settings [20,21,22]. PNE is an educational intervention in which the biological processes that underpin pain are explained, which serves as a tool to reduce pain itself [23]. The aim of this approach is to decrease the threat value of pain by reconceptualizing pain and increasing the patient’s knowledge of pain [24]. Currently there is no robust evidence for the efficacy of behavioral medicine for painful neuropathies, though this therapy shows promising results for some types of painful neuropathy [25].

Virtual Reality (VR) consists of a computer-generated simulation interactive environment that acts through the principles of immersion and presence. It generates a sense of being in the VR environment as place illusion, and the belief that events in VR are truly happening as plausibility illusion [26,27]. VR constitutes an enriched environment with augmented multiple sensory feedback (auditory, visual, and tactile VR enriched environments) that has already shown efficiency in reducing pain intensity, and improving functioning in different chronic pain settings, among which are patients with fibromyalgia, chronic low back and/or neck pain, complex regional pain syndrome and phantom limb pain [28,29]. Most VR interventions incorporate an attentional component yet, in chronic pain settings VR is mainly used as adjunct (due to the necessity of its immersive property [30]) to deliver psychological coping skills, improve mood or promote behavioral activities alongside the pharmacological treatment [31]. As such, VR could prove to be the delivery system for distraction as immediate pain relief, and PNE for long-term pain relief. The distracting character could also be applied as facilitator to enhance the process of falling asleep by inducing a relaxed state [32], as evidenced by promising reports on the efficacy of VR in promoting sleep [33,34,35]. In patients with non-malignant low back pain or fibromyalgia, sleep disturbance significantly improved after an immersive pain relief skills VR program, compared to sham VR with moderate effect sizes [36], or compared to an audio-only VR program [37]. Nowadays, several types of VR-assisted sleep interventions are available, among which are relaxation videos [33], mindfulness and hypnosis [34], and a virtual reality mental health training system with relaxation therapy, mindfulness meditation, and hypnotherapy [38], with beneficial outcomes on sleep parameters.

We hypothesize that, by providing an immersive VR experience to patients with PDPN an hour before bedtime, attention can be diverted from the pain condition, consequently leading to better sleep quality in patients suffering from PDPN. Moreover, the content of the VR experience focuses on PNE, known to have beneficial effects in patients with chronic pain [39,40]. Therefore, the aim of this study is to evaluate the efficacy of VR compared to usual care in improving sleep quality in patients suffering from PDPN.

## 2. Materials and Methods

### 2.1. Participants

In this study, male and female patients with diabetes mellitus with PDPN were invited to participate by the treating physician. The physician only asked patients to participate who had self-reported sleep problems. Inclusion criteria for recruitment were (1) aged between 18 and 70 years old, (2) able to speak Dutch or French, (3) continuing usual care regarding medication use 3 weeks prior to and during study participation, (4) diagnosis of PDPN for ≥6 months with confirmation by electromyography and (5) pain intensity score on the numeric rating scale ≥ 5/10 at baseline for average pain during the last 7 days. Exclusion criteria were (1) susceptibility to motion sickness or cyber-sickness and susceptibility to claustrophobia (based on self-reporting of patients), (2) shift workers and (3) patients with a history of seizure or epilepsy. Patients were included from January 2021 until July 2023.

Approval for the conduct of the study was obtained from the Ethics Committee of the Universitair Ziekenhuis Brussel, on 18 December 2019 (B.U.N. 143201942029). The study was conducted according to the principles laid down in the Declaration of Helsinki. The study was prospectively registered on ClinicalTrials.gov (NCT04325347) on 27 March 2020.

### 2.2. Study Design

**Figure 1:**
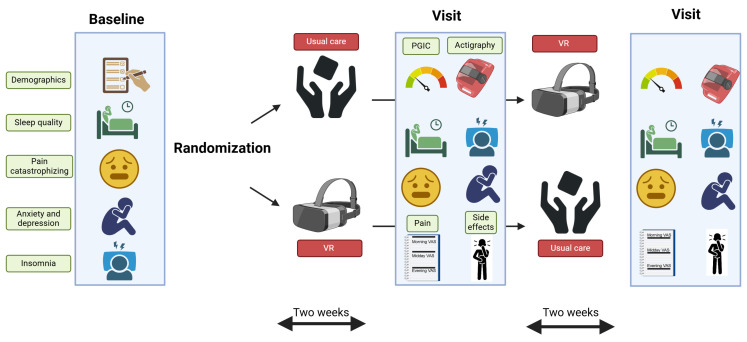
Study protocol. One baseline visit was conducted after which patients were randomized to either a 2-week period of Virtual Reality or usual care. After this, a cross-over took place to the other intervention. Outcome measurements were collected immediately after each intervention. Abbreviations: PGIC: patient global impression of change; VAS: Visual Analogue Scale; VR: Virtual Reality.

This was a single-center experimental proof of concept study investigating the effect of VR on overall sleep quality and number of awakenings in patients with PDPN. The study was conducted during three additional study visits at the hospital, with two weeks in between study visits (Figure 1). Patients did not have overnight hospital stays, and only visited the hospital for the 3 study visits of approximately 45 min each. Depending on the randomization, patients first received the experimental intervention for two weeks (i.e., VR intervention) or usual care (control intervention). After these two weeks, the alternative intervention was provided. A simple randomisation scheme based on patient numbers and the randomisation order that results from a computer-generated randomization procedure was stored in a sealed envelope. The study coordinator prepared separate envelopes for every patient before the start of the trial, which were opened by the investigator in the presence of the patient, after conducting the baseline assessment. Patients and investigators were not blinded for the study intervention, while the study coordinator was blinded.

During the baseline visit and the two subsequent visits, patients were asked to complete several questionnaires in an electronic format (with snapshot evaluations), received a pain intensity diary for the upcoming two weeks and received an actigraphy device. Actigraphy data were collected with continuous data registration during both interventions, as was pain intensity, with daily pain reporting using a pain diary during the interventions (i.e., ecological momentary assessment).

### 2.3. Outcome Measurements

During the first visit, patient’s demographics were collected (age, sex, pain duration and medication use). The primary outcome measurement consisted of an evaluation of sleep parameters measured with the Actiwatch and, more specifically, sleep efficiency and number of awakenings during the night. These parameters were chosen to provide a global overview of the quality of sleep, which is preferred above sleep quantity for assessing sleep [41]. The Actiwatch spectrum plus (Philips Respironics, Inc., Murrysville, PA, USA) was used to collect objective sleep quality parameters with reasonable reliability and validity in normal individuals with relatively good sleep patterns [42]. This electronic device is similar in size to a wristwatch and records physical movement with an accelerometer. It automatically collects data for seven sleep quality parameters: wake after sleep onset percentage, sleep onset latency, actual sleep percentage, mean night-time activity, fragmentation index, number of wake bouts, and sleep efficiency. Participants received the instruction to wear the activity monitors continuously (day and night) on their nondominant wrist. The average values of all sleep variables measured during two weeks by the actigraphy were used in the statistical analyses.

As secondary outcome measurements, patients completed several questionnaires during the hospital visits to evaluate sleep quality, insomnia, pain catastrophizing, anxiety, depression, and impression of change. Subsequently, patients were asked to report any side effects they experienced with the VR application through one open question. Finally, a VAS pain diary was completed with three measurements each day during the length of the study to measure pain intensity.

Perceived sleep quality was measured with the Pittsburgh Sleep Quality Index (PSQI). This questionnaire consists of 19 self-rated questions and 5 questions for the partner. The PSQI is the most widely used assessment of subjective sleep quality and contains 7 different sleep related components: sleep quality, latency, duration, habitual sleep efficiency, sleep disturbance, use of hypnotics and daytime functioning [43]. The self-rated questions were combined to provide scores from 0 to 3 on each of the 7 subcomponents. A score of 0 indicates no difficulties, while a score of 3 indicates difficulties more than three times a week. The subcomponent scores are then combined into a total score with a range from 0 to 21. A meta-analysis concluded that the PSQI has a strong reliability and validity [44]. A total score higher than 5 is considered an indicator of poor sleep quality [43]. Patients were instructed to take the time frame of the past two weeks into account for this questionnaire.

The Insomnia Severity Index (ISI) is a 7-item self-report questionnaire assessing the nature, severity, and impact of insomnia. The dimensions that were evaluated are severity of sleep onset, sleep maintenance, and early morning awakening problems, sleep dissatisfaction, interference of sleep difficulties on daytime functioning, noticeability of sleep problems by others, and distress caused by sleep difficulties. A 5-point Likert scale is used to rate each item (e.g., 0 = no problem; 4 = very severe problem), yielding a total score ranging from 0 to 28. The total score is interpreted as follows: absence of insomnia (0–7); sub-threshold insomnia (8–14); moderate insomnia (15–21); and severe insomnia (22–28) [45].

The Pain Catastrophizing Scale (PCS) is a self-reported questionnaire used to assess catastrophic thoughts or feelings accompanying previously experienced pain [46,47]. It consists of 13 items that evaluate 3 subscales of catastrophizing: rumination, magnification and helplessness on a 5-point Likert scale [46,47]. A total score > 30 represents a clinically significant level of pain catastrophizing. The PCS factor scales are valid and reliable in chronic pain [46,47,48].

The Hospital Anxiety and Depression Scale (HADS) is a questionnaire to assess anxiety and depressive symptoms and consists of 14 items, 7 items for the anxiety subscale (HADS Anxiety) and 7 for the depression subscale (HADS Depression). Each item is scored on a response-scale with four alternatives ranging between 0 and 3. After adjusting for 6 items that are reverse scored, all responses are summed to obtain the two subscales. Recommended cut-off scores are 8–10 for doubtful cases and ≥11 for definite cases [49]. The HADS was found to perform well in assessing the symptom severity of anxiety disorders and depression in both somatic, psychiatric and primary care patients and in the general population [50].

The Patient Global Impression of Change (PGIC) evaluates all aspects of patients’ health and assesses if there has been an improvement or decline in clinical status through a 7-point Likert scale.

Additionally, patients were also asked to report any side effects that they experienced with the VR application through an open question.

All patients completed a VAS pain diary with three measurements each day (average VAS score in the morning, midday, and evening) throughout the duration of the interventions. Scores were collected on a 10 cm horizontal line and expressed in mm. A score of 0 represented no pain at all, whereas a score of 100 represented the worst imaginable pain. The VAS is easy to use and is considered to provide reliable measurements of pain intensity [51].

### 2.4. Interventions

In the usual care intervention, patients continued their normal evening routine and did not receive any additional interventions. In the experimental intervention, patients were instructed to use the PICO VR goggle in their home environment for 14 days one hour before bedtime for about 20 min. The time frame of one hour before bedtime was chosen to provide enough distraction from pain before going to bed, while still being suitable for patient education. The commercially available virtual reality game Reducept (Reducept, Leeuwarden, The Netherlands) was played as psychological VR intervention for treating chronic pain. This application was chosen since it provides an environment that allows distraction and relaxation on the one hand, while also providing PNE. In this game, the patient travels with a spaceship throughout the body, i.e., from the nerves, over the spinal cord to the brain [52,53]. The application was developed to provide education about the mechanisms of pain, i.e., the cognitive, emotional, and behavioral processes related to pain and the pain sensation, and consists of five different modules [52,53]. In addition to PNE, the user plays several serious games (also called educational games) stimulating the visual, and auditory senses to manage chronic pain through distraction and relaxation and, as such, incorporating elements of cognitive behavioral therapy, mindfulness, and acceptance and commitment therapy [52,53]. A fixed scheme (i.e., which session to follow per day) was provided to the patients, whereby modules and games were mixed with each other, thereby fulfilling the logical order of the application. A written manual was provided to every patient containing the steps with which to control the VR goggle and to initiate the Reducept app. Additionally, during the study visit before the VR condition, the researcher made the patient comfortable in using the VR goggles.

### 2.5. Statistical Analysis

No formal sample size calculation was performed for this proof of concept study due to the lack of existing data on sleep parameters in patients with PDPN. The target sample for this study was to include 20 patients. Statistical analyses were performed using R studio Version 2022.07.2 (R version 4.4.0, Vienna, Austria.). All parameters were first checked for normality. Wilcoxon tests were applied to compare differences in primary outcome measurements (sleep efficiency and number of awakenings) between the two weeks, with and without VR. For secondary outcome measurements, the same test statistic was used. For PSQI, PCS, HADS and PGIC, relative difference scores were calculated between baseline scores and scores after the intervention, whereafter the difference scores were compared with Wilcoxon tests between both interventions. No imputation strategies were applied for missing data.

## 3. Results

### 3.1. Descriptive Statistics

In total, eight patients were included in this study, though the primary outcome was not collected for one patient due to not correctly and not continuously wearing the Actiwatch device. This patient was excluded from the study, whereby this study reports on data from seven patients. Recruitment was slower than anticipated due to the COVID-19 pandemic (anticipated study start in December 2019) and language restrictions. The first patient was recruited on 27 January 2021 and the last patient on 26 June 2023. Four males and three females took part in the study, with a median age of 61 (Q1–Q3: 57.5–66.5) years. The median BMI of included patients was 31.99 (Q1–Q3: 29.85–34.26) kg/m^2^. Three patients first received usual care intervention, while four patients first received VR before the cross-over. At baseline, all patients had a total PSQI score higher than 5, indicating poor sleep quality. Individual patient demographics are presented in Table 1.

### 3.2. Primary Outcome Measurements: Sleep Parameters

For sleep efficiency, median values of 81.91% (Q1–Q3: 75.27–84.40%) were revealed during the usual care and 79.75% (Q1–Q3: 75.20–84.56%) during VR. No statistically significant difference was revealed between both interventions (V = 12, *p* = 0.81). For number of awakenings, median values of 44 (Q1–Q3: 29.48–65.97) during usual care did not statistically differ from median values of 40.88 (Q1–Q3: 31.36–52.83) during VR (V = 110, *p* = 1). For time spent in bed, sleep time, sleep latency and WASO, no statistically significant differences were revealed (Table 2).

### 3.3. Secondary Outcome Measurements: Clinical Questionnaires

After statistically comparing sleep quality, insomnia, pain catastrophizing, anxiety, depression and pain intensity scores between both interventions, no significant differences were revealed. Median values and test statistics are reported in Table 2. As a sensitivity analysis, results before the cross-over were analysed separately, with results pointing to similar conclusions (Supplementary Materials Table S1).

All patients were capable of handling VR goggles. With respect to side effects reported after VR, only one patient reported a finding, i.e., a self-reported decrease in blood sugar level (causality unclear and not further explored). In terms of impression of change after the interventions, one patient reported a minimal improvement after usual care, five reported no change and one patient reported a worsening. After VR, three patients reported that their clinical status had much improved, three reported that their status had minimally improved and only one patient reported no change. Between both groups, a statistically significant difference was found (V = 21, *p* = 0.03).

## 4. Discussion

A 2-week period of VR, providing PNE and distraction, did not result in increased sleep efficiency or fewer awakenings compared to usual care in patients with PDPN. Sleep quality, insomnia, pain catastrophizing, anxiety, depression and pain intensity scores did not differ between usual care and VR. Only on the PGIC, used to assess improvement or decline in clinical status, did more patients improve after VR compared to after usual care treatment. This proof of concept demonstrated that VR resulted in minimal and mild side effects, thereby confirming the safety and feasibility of embedding VR in home settings.

These results indicate that patients subjectively experience an improvement, yet this is not substantiated by neither self-reported nor objective measurements. PGIC represents a clinically relevant tool to assess the perceived impact of disease management [54]. Despite significant correlations between impression of change and functionality or pain measures [55], it was previously denoted that this outcome measure should not be considered in isolation but within the global clinical context [54,56]. It may be hypothesized that the improvement in PGIC is related to a placebo effect, driven by heterogeneous underlying theories, among which are expectation and the patient–physician relationship [57]. Expectation, or anticipated future outcomes, posits that positive and conscious expectations will cause beneficial outcomes [58], and is denoted as the dominant theory regarding placebo effects [59]. Positive expectations towards VR, combined with the clinical encounter during the patient–physician relationship (i.e., a complex assemblage of explicit behaviours and embodies and implicit non-verbal cues) during the recruitment phase of this study [57], could explain the positive effects on PGIC. 

This negative results in this study could be attributed to fourreasons. Firstly, despite the fact that up to 30% of patients with diabetic polyneuropathy will develop PDPN [4,5], the efficacy of PNE in patients with diabetes mellitus is rarely investigated. Secondly, Reducept as a web application was developed some years ago, yet research with Reducept in the context of improving sleep quality is still lacking. There are promising results with Reducept in decreasing pain in chronic low back pain patients [52]. Additionally, a pilot study in patients with chronic low back pain revealed a positive effect on pain intensity scores after 10 min of daily activity on the application for 4 weeks [60]. Relying on a single-case experimental design in eight patients with chronic low back pain, Reducept was able to reduce low back pain intensity, although the clinical relevance was small, whereas no robust effect was found for pain catastrophizing, psychological complaints, fear of movement, pain coping or quality of life [52]. An online survey of 265 Dutch primary care physiotherapists denoted that 7% of the physiotherapists used VR in the treatment of patients with chronic pain, whereby Reducept was used by 50% of the therapists (ranked in place 1) [61].

Thirdly, it may also be suggested that our time frame of 2 weeks is too short to induce alterations in both self-reported and objective measurements. However, providing PNE already revealed beneficial effects after 2 h of education [62]. Furthermore, actigraphy measures and self-reported sleep questionnaires previously revealed adaptations after two week interventions [35,63], whereby this time frame seems plausible to detect intervention differences.

Finally, it may be possible that PNE is not yet suitable for incorporation into VR applications and should be further adapted towards this new framework, compared to classical and individual real-life education. Recent RCTs concluded that PNE in a VR setting may be acceptable and feasible for patients with chronic low back pain [64] and that VR neuroscience-based therapy showed preliminary efficacy in reducing pain and improving overall functioning in chronic back pain patients [65], wherefore further comparative explorations regarding the efficacy of VR versus real-life PNE in different patient populations are currently highly needed. In verbal PNE administration modes, two sessions of up to 1 h are already enough to see positive results for quality of life, symptoms of kinesiophobia and pain catastrophizing [62], wherefore the negative results in this study are presumably not related to the duration of providing PNE (i.e., 20 min for 14 days). When taking into account these four reasons, it becomes clear that more research is needed to further explore the efficacy of PNE both in terms of suitable populations (e.g., PDPN) and in terms of delivery method (i.e., verbal versus VR versus written). Additionally, Reducept is commonly used in clinical practice, but efficacy of this VR application, especially in terms of improving sleep quality, should be further investigated.

Combining all findings, it may be possible that PNE as stand-alone treatment is not suitable for patients with PDPN and that combinations with other lifestyle interventions are necessary (among which are dietary interventions or sleep management) [66]. An ongoing three arms trial in 360 patients with chronic low back pain is evaluating the efficacy of (1) skills-based VR (incorporating cognitive behavioural therapy, mindful meditation, and physiological biofeedback therapy using embedded biometric sensors); (2) distraction-based VR (360-degree immersive videos designed to distract users from pain); and (3) sham VR (non-immersive videos) over up to 56 days and with several outcome measures, among which are sleep disturbances [67]. In case this study reveals positive effects of skills-based VR, it could be suggested that the currently used Reducept application should be combined with other treatments to obtain effects on sleep quality.

In line with the recommendations to provide multimodal treatment for patients with diabetic polyneuropathy [68], this proof of concept study incorporated a functional primary outcome measurement, namely sleep quality. Additionally, despite the pilot character, both interventions were randomized with a cross-over after two weeks to ensure that all patients could benefit from the VR. This study also has limitations that should be considered when interpreting the study results. Firstly, this study might have benefitted from a larger sample size for sufficient power to answer the research question, wherefore this proof of concept study merely serves as a starting point to provide pilot data to substantiate sample size calculations for larger comparative trials in this population, whereby intervention compliance should be carefully monitored, as was not the case in this study. Additionally, this study only evaluated patients with PDPN, whereby the use of VR to improve sleep quality should also be further evaluated in other chronic pain conditions. In terms of outcome measures, actigraphy and pain intensity were compared between both interventions, whereby an additional baseline visit was not conducted to decrease patient burden. There was no wash-out period between both interventions, since it was assumed that the distraction element of the VR condition was crucial for enhancing sleep efficiency. An analysis only based on data before the cross-over phase confirmed the results, but future studies should evaluate the implementation of a wash-out period. Furthermore, the intervention was provided one hour before bedtime, yet further research is needed as to whether this is the best time frame to ensure distraction and education. Finally, no control group was added to this study since the usual care group was used as reference for the VR condition.

## 5. Conclusions

In patients with PDPN, the use of VR for a two-week period does not improve sleep quality, psychological measures or pain intensity. However, compared to the start of the study, patients subjectively reported a positive change after VR.

## Figures and Tables

**Table 1:** Individual patient demographics. Abbreviations. F: female; M: male; y: years.

Patient ID	Age	Sex	Pain Duration	BMI (kg/m^2^)	Medication Use
1	57	M	20y	36.23	Apidra, Insuman Basal, Nobiten, Metformax, Neurontin, Zocor, Redomex, Coveram
2	73	F	3y	31.99	Metformine Mylan, Coversyl, Simvastatin sandoz, Neurontin, Nexiam, Redomex
3	68	M	13y	31.96	Metformine Mylan, Nobiten, Lyrica, Sildenafil Apotex, INS Actrapid, Asaflow, Zocor
4	65	M	18y	32.28	Lyrica, Redomex, Rivotril, Olmetec, Nebivolol, Zanidip, Crestor, Metformax, Unidiamicron, Tribvit, Tardyferon
5	58	F	2y	25.71	Amlodipine, Medformine, Lantus, Lyrica, Stilnoct
6	61	M	4y	27.73	Novoprifit, Lantus, Metformax
7	56	F	31y	39.45	Ms Contin, Ms Direct, Medrol, Arcoxia

**Table 2:** Actigraphy and clinical questionnaires during usual care and VR.

Outcome Measurement	Baseline	Usual Care	VR	Wilcoxon Test	N
Actigraphy					
Sleep efficiency (%)		81.91 (75.27–84.40)	79.75 (75.20–84.56)	V = 12, *p*-value = 0.81	7
Number of awakenings (count)		44.00 (29.48–65.97)	40.88 (31.36–52.83)	V = 11, *p*-value = 1	6
WASO (min)		34.69 (33.64–62.42)	39.23 (33.27–53.87)	V = 10, *p*-value = 1	6
Time in bed (min)		498.1 (489.8–534.2)	510.4 (499.8–523.2)	V = 16, *p*-value = 0.81	7
Sleep time (min)		417.4 (373.3–428.9)	415.5 (400.1–432.6)	V = 9, *p*-value = 0.47	7
Sleep latency (min)		26.00 (18.98–38.56)	29.25 (21.86–54.07)	V = 10, *p*-value = 1	6
Questionnaires					
PSQI (/21)	11 (10–14)	9 (7.5–10)	9 (7.5–10.5)	V = 10, *p*-value = 1 *	7
ISI (/28)	10 (9–15)	10 (8–13)	7 (6–8.5)	V = 7, *p*-value = 0.30 *	7
PCS (/52)	20 (17.5–28.5)	23 (12.5–24.5)	20 (19–28.5)	V = 18, *p*-value = 0.55 *	7
HADS anxiety (/21)	8 (5.5–9)	8 (3–9)	5 (3.5–6.5)	V = 4.5, *p*-value = 0.25 *	7
HADS depression (/21)	8 (3–11)	6 (3.5–7.5)	6 (2–8)	V = 14, *p*-value = 1 *	7
PGIC		Minimally improved: 1 No change: 5 Worse: 1	Much improved: 3 Minimally improved: 3 No change: 1	V = 21, *p*-value = 0.03	7
Side effects			No: 6 Self-reported decrease in blood sugar level: 1		7
Pain intensity morning (/100)		37.3 (32.6–38.5)	26.2 (22.0–41.3)	V = 11, *p*-value = 0.44	5
Pain intensity midday (/100)		56.7 (39.7–58.1)	43.6 (34.9–49.7)	V = 10, *p*-value = 0.62	5
Pain intensity evening (/100)		66.9 (47.2–75.6)	59.0 (41.0–67.1)	V = 12, *p*-value = 0.31	5

*: hypothesis testing was performed on the difference between baseline and VR condition versus baseline and usual care condition. Abbreviations: min: minutes; N: number of patients; VR: Virtual Reality.

## Data Availability

All data generated or analyzed during this study are included in the published article. The datasets are available from the corresponding author on reasonable request.

## Supplementary Materials

The following supporting information can be downloaded at: https://www.mdpi.com/article/10.3390/jcm13237163/s1, Table S1: Study results with data before cross-over.
