# Supplementary material for: Virtual Reality to Improve Sleep Quality in Patients Suffering from Painful Diabetic Polyneuropathy: A Proof of Concept Study
Source: J Clin Med. 2024 Nov 26;13(23):7163. doi: 10.3390/jcm13237163 (PMC11642838; doi:10.3390/jcm13237163)
Supplement: Supplementary file 1 [file jcm-13-07163-s001.zip › jcm-3272638-supplementary.pdf]

Supplementary Material I

Outcome measurement	Usual care	VR	Wilcoxon test
Actigraphy			
Sleep efficiency (%)	83.23<br>(80.17 – 84.40)	75.20<br>(71.87 – 80.33)	W = 8, p-value = 0.63
Number of awakenings (count)	27.93<br>(22.36 – 40.90)	47.83<br>(40.88 – 61.19)	W = 2, p-value = 0.4
WASO (min)	33.54<br>(24.75 – 53.30)	39.54<br>(34.97 – 55.85)	W = 4, p-value = 1
Time in bed (min)	498.1<br>(496.7– 506.6)	518.4<br>(510.4 – 531.3)	W = 2, p-value = 0.23
Sleep time (min)	417.4<br>(398.8 – 422.4)	426.2<br>(403.4 – 441.5)	W = 5, p-value = 0.86
Sleep latency (min)	30.55<br>(26.00 – 35.89)	27.79<br>(23.84 – 58.37)	W = 5, p-value = 1
Questionnaires			
PSQI (/21) *	0.18<br>(0.09 – 0.34)	0.24<br>(0.07 – 0.40)	W = 6, p-value = 1
ISI (/28) *	0.00<br>(-0.12 – 0.17)	0.30<br>(-0.04 – 0.60)	W = 3.5, p-value = 0.47
PCS (/52) *	0.22<br>(-0.21 – 0.61)	0.08<br>(0.02 – 0.20)	W = 7, p-value = 0.86
HADS anxiety (/21) *	0.00<br>(-0.17 – 0.00)	0.23<br>(0.09 – 0.42)	W = 1, p-value = 0.10
HADS depression (/21) *	0.15<br>(0.08 – 0.18)	0.54<br>(-0.15 – 0.87)	W = 3, p-value = 0.4
PGIC	No change: 3	Much improved: 2<br>Minimally improved: 2	W = 12, p-value = 0.04
Pain intensity morning (/100)	48.9<br>(34.5 – 63.4)	26.2<br>(24.7 – 31.4)	W = 4, p-value = 1
Pain intensity midday (/100)	43.1<br>(33.2 – 52.9)	43.6<br>(36.2 – 47.3)	W = 5, p-value = 0.8
Pain intensity evening (/100)	65.4<br>(56.3 – 74.5)	51.1<br>(32.4 – 65.8)	W = 6, p-value = 0.53

Table: Study results with data before cross-over. *: difference score between baseline and condition.
